# Mechanism of *Echinochloa crus-galli* Resistance to the ALS-Inhibiting Herbicide Pyrazosulfuron-ethyl in China

**DOI:** 10.3390/plants15111611

**Published:** 2026-05-24

**Authors:** Qing Liu, Rongxue Zhang, Linjing Sun, Xin Lu, Gaoping Xu, Hui Tong, Binglei Zhang, Xuejun Liu, Shengli Du

**Affiliations:** 1 College of Life Sciences, Nankai University, Tianjin 300071, China; 2 Modern Agricultural Science and Technology Research Institute, Tianjin Agricultural University, Tianjin 300392, China; 3 Tianjin Key Laboratory of Crop Genetics and Breeding, Tianjin Crop Research Institute, Tianjin 300392, China; 4 Cucumber Research Institute, Tianjin Academy of Agricultural Sciences, Tianjin 300192, China; 5 State Key Laboratory of Vegetable Biobreeding, Tianjin 300192, China

**Keywords:** *E. crus-galli*, herbicide resistance, ALS inhibitors, mutation, acetolactate synthase, TSR mechanisms

## Abstract

Rice (*Oryza sativa* L.) is a staple food crop, feeding more than 3.5 billion people. With the increasing demand for food in the 21st century, weed infestation poses the most significant biotic threat to global food security, and herbicides remain the most effective and economic way to manage it in field. However, weeds can rapidly adapt under herbicide selection pressure due to their high competitiveness, rapid growth, and reproductive capacity. Hence, we collected *Echinochloa crus-galli* populations from Heilongjiang and Hebei provinces in China and investigated their resistance mechanisms to pyrazosulfuron-ethyl (PSE), a sulfonylurea herbicide that inhibits acetolactate synthase (ALS). Dose–response experiments confirm that the resistant (R) population exhibits 52.9-fold resistance to PSE compared with the susceptible (S) population. Inhibitor bioassays with malathion and NBD-Cl, together with ALS activity assays, ALS gene sequencing, and molecular docking, collectively suggest that resistance is strongly associated with the ALS Trp-574-Leu target-site substitution, with a possible additional contribution from enhanced herbicide metabolism. However, because the S and R populations originate from geographically distinct locations, some of the observed physiological and molecular differences may also reflect inherent population variation. Specifically, the ALS W574L substitution is predicted to reduce key interactions between ALS and PSE. This study provides valuable evidence for the risk of PSE resistance evolution in *E. crus-galli* and elucidates the molecular mechanism conferring resistance to ALS inhibitors.

## 1. Introduction

Direct-seeded rice systems have expanded rapidly in Heilongjiang Province—China’s largest direct-seeded rice region—where pyrazosulfuron-ethyl (PSE), a sulfonylurea herbicide targeting acetolactate synthase (ALS), has been used extensively for *Echinochloa crus-galli* (barnyardgrass) control for over 15 years [1,2,3,4]. This annual grass aggressively competes with rice for nutrients, light, and water, causing substantial yield losses [5,6]. However, prolonged reliance on PSE has imposed intense selection pressure, leading to the emergence of resistant *E. crus-galli* populations and threatening the sustainability of rice production in this region.

ALS inhibitors, including sulfonylureas like PSE, are among the most widely adopted herbicides due to their low application rates, broad-spectrum efficacy, and high crop safety. However, their extensive use has resulted in the evolution of resistance in numerous weed species [7].

The mechanisms of resistance are classified into target-site resistance (TSR) and non-target-site resistance (NTSR) [8,9,10]. TSR arises from amino acid substitutions in the ALS enzyme that reduce herbicide binding [11]. To date, at least seven conserved residues—Ala-122, Pro-197, Ala-205, Asp-376, Trp-574, Ser-653, and Gly-654—have been identified in resistant weed populations, with different substitutions conferring distinct cross-resistance spectra across the five chemical classes of ALS inhibitors [12,13]. Notably, these mutations fall into two functional categories: those that confer broad cross-resistance to multiple chemical classes, such as Trp-574-Leu, and those that provide class-specific resistance, such as certain Pro-197 substitutions [11]. The rapid enrichment of these resistance alleles in the field is facilitated by a high initial mutation frequency (10^−6^ to 10^−8^), simple and dominant nuclear inheritance allowing transmission by both seed and pollen, and the negligible fitness cost of resistance alleles in the absence of herbicide selection [11].

In addition, previous study reveals that substitutions at Trp-574 alter the herbicide-binding pocket sufficiently to exclude multiple chemical classes, whereas substitutions at Pro-197 specifically disrupt the binding of sulfonylureas [14]. In parallel, NTSR—primarily enhanced metabolic detoxification mediated by cytochrome P450 monooxygenases—has been recognized as an equally important resistance mechanism in several weed species, although the specific resistance-endowing genes were yet to be identified [14]. In *Echinochloa* species, such metabolic resistance, often involving both P450s and glutathione *S*-transferases, has been increasingly documented and confers cross-resistance to ALS inhibitors, ACCase inhibitors, and synthetic auxin herbicides [6,15].

Heilongjiang Province, a major direct-seeded rice region in China, faces serious weed management challenges, including synchronous emergence of rice and weeds, high weed species diversity, and increasing herbicide resistance, all of which have substantially reduced rice yields. In this study, we selected PSE as a representative ALS-inhibiting herbicide and evaluated the sensitivity of an *E. crus-galli* resistant (R) population from rice fields in the Heilongjiang Province, Northeastern China. This study aimed to (1) determine the resistance levels of PSE in R and sensitive (S) populations; (2) obtain the DNA sequence of the ALS and identify key amino acids substitutions contributing to PSE resistance in R populations; and (3) validate the mechanism of resistance to ALS-inhibiting herbicides in *E. crus-galli*. These findings contribute to a better understanding of ALS-mediated herbicide resistance on weed management in Heilongjiang province.

## 2. Results

### 2.1. Sensitivity of E. crus-galli to PSE

**Figure 1:**
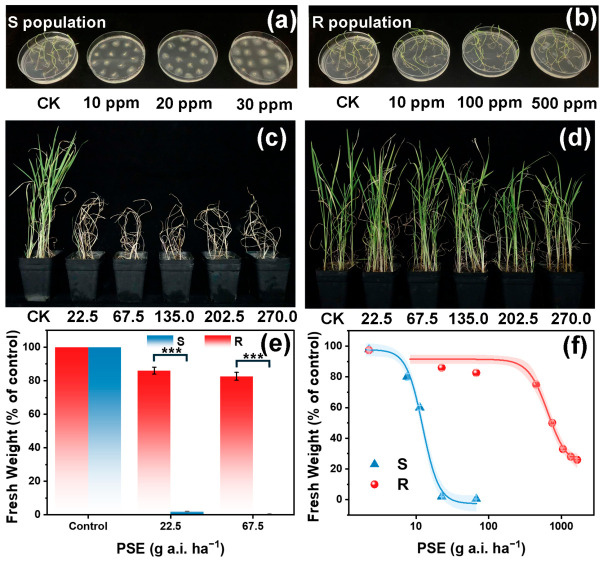
Effect of PSE on seed germination and seedling growth for S (**a**) and R (**b**) populations of *E. crus-galli*. Whole-plant herbicide sensitivity bioassays of S and R populations. Growth response to PSE of S (**c**) and R (**d**) under dosages of 0, 22.5, 67.5, 135.0, 202.5 and 270.0 g a.i. ha^−1^. (**e**) The relative fresh weight of S and R populations under dosages of 0, 22.5 and 67.5 g a.i. ha^−1^. (**f**) Dose–response of R and S populations to PSE. Fresh weights were recorded after 21 days of treatment. Each point represents the mean ± standard error (SE) of three replicates. Asterisks indicate significant differences between S and R populations within each dose (*** *p* < 0.001, two-way ANOVA followed by Tukey’s HSD test).

The Petri dish germination assay was first conducted to explore the effect of PSE on seed germination and seedling growth (Figure 1a,b). For the S population, both germination and subsequent growth were completely inhibited when the PSE concentration reached or exceeded 10 ppm. Notably, the R population germinated and grew normally even at 500 ppm PSE, with no discernible difference from the control, demonstrating a high level of resistance to PSE in the R population.

To further evaluate the resistance of the R population and the S population to PSE of Heilongjiang province, three-leaf *E. crus-galli* plants were treated with PSE at gradient rate; then the phenotypes of aboveground tissues were measured. As expected, the R population displayed high resistance to PSE [15]. While the S population was effectively controlled at the recommended field rate (22.5 g a.i. ha^−1^), the same rate failed to control the R population. Even at 12 times the recommended rate (270 g a.i. ha^−1^), the R population was not effectively controlled. (Figure 1c,d). As shown in Figure 1e, the R population had significantly higher fresh weight compared to the S population after PSE treatment. At the recommended field rate of PSE, the fresh weight of the S population was reduced to 2.2% of the control, whereas the R population retained 86.0% of the control. Consequently, the fresh weight of the R population was approximately 39-fold higher than that of the S population under this treatment. At three times the recommended field rate (67.5 g a.i. ha^−1^), PSE reduced the fresh weight of the S population to an undetectable level (complete inhibition), whereas the R population showed only a 3% reduction relative to its own untreated control.

To quantify the resistance of S and R populations to PSE, the whole-plant dose–response experiment was employed [15]. The results showed that the GR_50_ of PSE in the R and S populations are 651.76 g a.i. ha^−1^ and 12.32 g a.i. ha^−1^, respectively (Table 1). The R population exhibited high resistance, with an RI of 52.9 (extra sum-of-squares F-test, F = 227.95, *p* < 0.0001; Table 1). An extra sum-of-squares F-test confirmed that the GR_50_ value of the R population (651.76 g a.i. ha^−1^) was significantly higher than that of the S population (12.32 g a.i. ha^−1^; F = 227.95, *p* < 0.0001; Table 1). Similarly, the dry weight, survival rate and plant height of the R population confirmed greater PSE tolerance in the R population relative to the S population (Figure 1f and Figures S1–S3). Clearly, the substantial disparity in PSE tolerance between the S and R populations suggests the evolution of resistance to this ALS-inhibiting herbicide in the R population. These findings provide a critical framework for subsequent investigations into the mechanistic basis of differential herbicide tolerance. It should be noted that the S and R populations were collected from different provinces; while the S population has no history of ALS-inhibiting herbicide exposure, inherent geographical variation may account for some of the phenotypic differences observed.

### 2.2. Sequencing and Sequence Analysis of the ALS Gene

**Figure 2:**
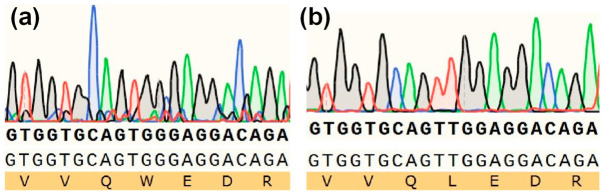
Comparison of ALS gene sequences between S (**a**) and R (**b**) populations. The color code for nucleotide bases is as follows: green for adenine (A), red for thymine (T), blue for cytosine (C), and black for guanine (G).

To investigate the role of TSR mechanism in PSE resistance, the ALS gene was sequenced and compared between the S and R populations [16]. Sequence alignment analysis confirmed the presence of a nonsynonymous nucleotide mutation in the R population compared to the S population (Figure 2a,b). This mutation resulted in a W574L amino acid substitution (TGG to TTG) in the R population. The W574L substitution (TGG→TTG) was detected in all 10 individual plants sequenced from the R population, with no other resistance-associated substitutions observed at the remaining eight conserved ALS positions (Ala-122, Pro-197, Ala-205, Phe-206, Asp-376, Arg-377, Ser-653, and Gly-654). Other than this mutation, the nucleotide sequences of the S and R populations were identical at all known resistance-conferring sites (Tables S2 and S3).

Though this indicates a high mutation frequency in the sampled individuals, further genotyping of more field-collected individuals is needed to confirm if the mutation is fixed across the entire field population, avoiding the implication of genetic uniformity for this mutation in the whole population. Based on previous studies, three primer pairs conventionally used for ALS resistance analysis in Echinochloa crus-galli were selected, covering key target-site resistance mutation sites of ALS1, ALS2 and ALS3 [17]. Sanger sequencing of the amplicons confirmed they shared up to 99.58% homology with ALS1; sequence differences from ALS2 and ALS3 were only in non-essential regions outside documented resistance-related mutation sites.

As *E. crus-galli* is polyploid, potential resistance mutations not amplified by these primers may remain undetected.

Notably, the W574L substitution is a well-characterized ALS mutation endowing resistance to ALS inhibitor chemical families, indicating that the herbicide resistance of *E. crus-galli* populations might be conferred by this amino acid (W574L) [18].

### 2.3. The Effect of Cytochrome P450 (CYP450s) and Glutathione S-Transferases (GSTs) Inhibitors for PSE Resistance

To further evaluate the contributions of CYP450s and GSTs to the detoxification of PSE in *E. crus-galli*, malathion and NBD-Cl, typical inhibitors of CYP450s and GSTs activity, were employed to validate PSE resistance mode of action, respectively [6].

**Figure 3:**
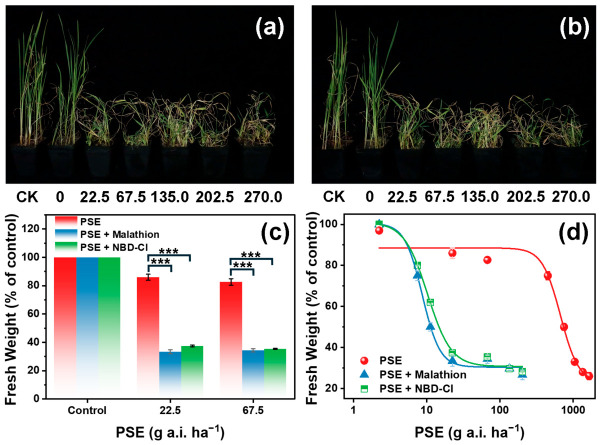
Effect of metabolic inhibitors on PSE resistance in the R population. Growth response to PSE with malathion (**a**) and PSE with NBD-Cl (**b**) under PSE dosages of 0, 22.5, 67.5, 135.0, 202.5 and 270.0 g a.i. ha^−1^. (**c**) Relative fresh weight of R populations in response to PSE (0, 22.5, and 67.5 g a.i. ha^−1^) alone and in combination with malathion and NBD-Cl. (**d**) Dose–response of R population to PSE with malathion and PSE with NBD-Cl. Fresh weights were recorded after 21 days of treatment. Each point represents the mean ± SE of three replicates. Asterisks indicate significant differences compared to the untreated control (*** *p* < 0.001, one-way ANOVA followed by Dunnett’s test).

As shown in Figure 3a–c, treatment with malathion or NBD-Cl alone resulted in almost no change in growth compared to the untreated control (CK). However, a notable inhibition of growth was observed when either inhibitor was co-applied with PSE, even at the recommended field dose. The GR_50_ of the R population significantly decreased from 651.76 to 8.88 g a.i. ha^−1^ when PSE was combined with the CYP450 inhibitor malathion at 1000 g a.i. ha^−1^, corresponding to a 98.7% reduction (extra sum-of-squares F-test, *p* < 0.0001; Table 2). Similarly, treatment with NBD-Cl at 270 g a.i. ha^−1^ reduced the GR_50_ to 10.51 g a.i. ha^−1^, a 98.4% reduction (*p* < 0.0001; Table 2). These results suggest that metabolic resistance, potentially mediated by CYP450s and GSTs, may contribute to PSE resistance in the R population [7,19].

### 2.4. Analysis of ALS Enzyme Activities

**Figure 4:**
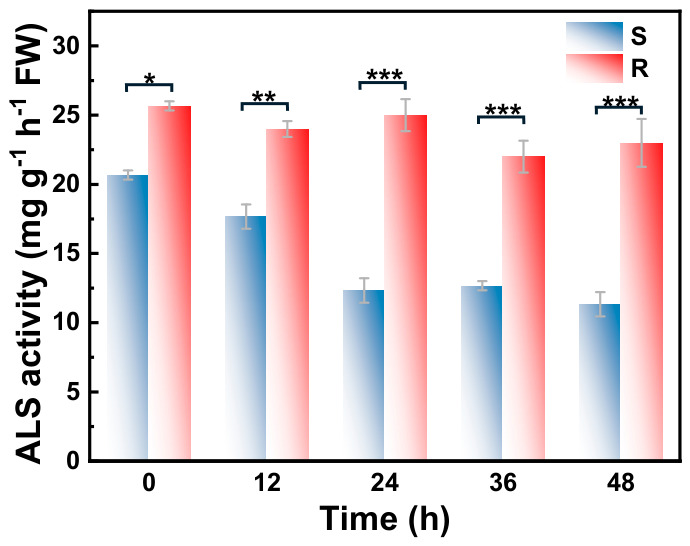
Effect of PSE (22.5 g a.i. ha ^−1^) on ALS activity in S and R populations. Values represent mean ± SE of three independent biological replicates. Data were analyzed by two-way ANOVA (population: F = 233.65, *p* < 0.001; time: F = 15.02, *p* < 0.001; population × time interaction: F = 6.31, *p* = 0.0019). Asterisks indicate significant differences between S and R populations at each time point by Tukey’s HSD test: * *p* < 0.05, ** *p* < 0.01, *** *p* < 0.001.

Given the above analysis of PSE resistance in *E. crus-galli*, ALS activity in both the S and R populations was measured according to previous studies [20]. Although ALS activity decreased in both populations at 12 h, the activity in the R population remained significantly higher than that in the S population (Figure 4). At 48 h post-treatment with PSE, ALS activity had declined from 20.67 ± 0.33 to 11.33 ± 0.88 mg g^−1^ h^−1^ FW in the S population, whereas it decreased only slightly from 25.67 ± 0.33 to 23.00 ± 1.73 mg g^−1^ h^−1^ FW in the R population (Figure 4).

Furthermore, two-way ANOVA revealed a significant population × time interaction (F = 6.31, *p* = 0.0019), as well as significant main effects of population (F = 233.65, *p* < 0.001) and time (F = 15.02, *p* < 0.001). Tukey’s HSD post-hoc test showed that ALS activity in the R population remained significantly higher than in the S population at all time points (Table 3). In the S population, ALS activity declined progressively from 20.67 ± 0.33 at 0 h to 11.33 ± 0.88 mg g^−1^ h^−1^ FW at 48 h. In contrast, ALS activity in the R population showed only a slight decrease, from 25.67 ± 0.33 at 0 h to 23.00 ± 1.73 mg g^−1^ h^−1^ FW at 48 h. ALS activity in the R population remained significantly higher than in the S population at all time points after PSE treatment, indicating that the mutant enzyme is less sensitive to inhibition. Notably, the baseline ALS activity (0 h) in the R population was 24.2% higher than in the S population (25.67 vs. 20.67 mg g^−1^ h^−1^ FW, *p* = 0.0295). This difference may reflect inherent genetic variation between the geographically distinct populations, in addition to any potential effects of the W574L substitution on enzyme activity.

### 2.5. Molecular Docking of ALS Isoforms with PSE and Structure Activity Relationships

**Figure 5:**
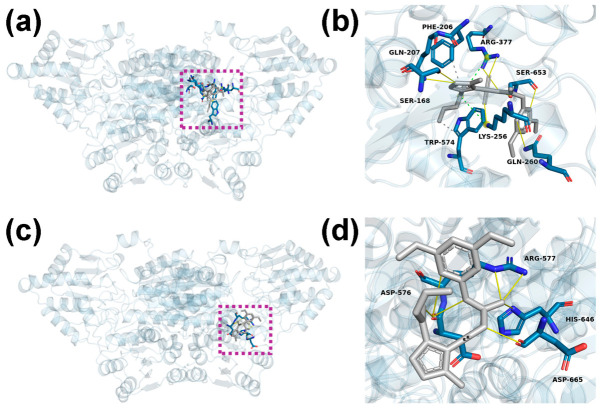
Molecular docking models of PSE with ALS from the S and R populations. (**a**) Overall binding pose of PSE on the molecular surface of the wild-type ALS (S population) and (**b**) close-up view of the predicted substrate-binding pocket. (**c**) Overall binding pose of PSE on the molecular surface of the W574L mutant ALS (R population) and (**d**) close-up view of the corresponding pocket. Intermolecular interactions predicted by docking are highlighted in yellow (hydrogen bonds), gray (hydrophobic interactions), and green (π-stacking).

To help elucidate the mechanism by which the W574L mutation confers PSE resistance, we constructed three-dimensional homology models of ALS from the S and R populations [21,22]. Molecular docking using AutoDock Vina was performed to evaluate the binding of PSE to both ALS variants. The optimal binding pose was selected based on binding energy ranking and subjected to visual analysis. The binding energy of PSE to the wild-type ALS was −6.61 kcal mol^−1^, whereas that to the W574L mutant was −4.61 kcal mol^−1^, indicating a considerably lower predicted affinity for the mutant. These in silico predictions are consistent with the resistance phenotype and provide a structural hypothesis, suggesting that the S population ALS may have a higher affinity for PSE than the R population variant. The docked conformations of PSE within the ALS active site cavities are illustrated in Figure 5.

As shown in Figure 5a,b, the wild-type ALS from the S population could favorably accommodate PSE at the active site. In the predicted binding pose, PSE is harbored within a pocket formed by eight residues. Residues Ser-168, Gln-207, Lys-256, Gln-260, Arg-377, and Ser-653 are predicted to form conventional hydrogen bonds with the ligand, while Phe-206 and Trp-574 could participate in hydrophobic interactions (Table S7). Additionally, Lys-256 and Arg-377 are predicted to form salt bridges with PSE. In the W574L mutant model, the substitution of tryptophan by leucine is predicted to alter the shape of the binding pocket, resulting in a predicted binding pose with fewer interactions (Figure 5c,d). In this model, only residual hydrogen bonding with Asp-576, Arg-577, His-646, and Asp-665 are observed (Table S7). Taken together, the in silico docking analysis provides a structural hypothesis consistent with the observed resistance phenotype: the W574L substitution disrupts key hydrophobic interactions and alters the shape of the herbicide-binding pocket, leading to a predicted 2.0 kcal mol^−1^ reduction in binding affinity. However, this prediction requires experimental validation through X-ray crystallography or site-directed mutagenesis to confirm the precise binding mode and functional consequences of the mutation [23].

## 3. Discussion

Synthetic chemical herbicides offer an efficient approach to weed management in rice production, providing economic and operational flexibility [21]. Although significant progress has been made in this field, the widespread applicability of chemical control is often constrained by factors such as geographical variation and herbicide selectivity. Since the commercialization of ALS-inhibiting herbicides in the 1980s, five major chemical classes—sulfonylureas (SU), imidazolinones (IMI), triazolopyrimidines (TP), pyrimidinyl thiobenzoates (PTB), and sulfonylamino-carbonyl-triazolinones (SCT)—have been widely used in Heilongjiang Province [24]. However, the evolution of herbicide resistance has become a widespread challenge resulting from the intensive and sustained use of these chemicals to meet the demands of burgeoning rice production. This resistance progressively diminishes the efficacy of herbicides in the field. Moreover, regional variation in herbicide selection practices further complicates weed management, exacerbating the difficulty of effective control. Therefore, a comprehensive understanding of the mechanisms underlying herbicide resistance is imperative for developing sustainable management strategies in agricultural systems.

PSE, a novel SU herbicide targeting ALS, offers significant potential for weed management owing to its high potency, broad herbicidal spectrum, low mammalian toxicity, and favorable environmental profile. Here, we identified an *E. crus-galli* population from Heilongjiang Province that exhibits unexpectedly high resistance to PSE. Dose–response assays revealed a GR_50_ of 651.76 g a.i. ha^−1^ for this R population, representing a 52.9-fold resistance level compared to S population from Hebei Province (Table 1). This resistance level falls within the range of previous observations for ALS inhibitors in *E. crus-galli* populations across China. A susceptibility monitoring study covering 30 *E. crus-galli* populations in the Mid-Lower Yangtze rice region reported penoxsulam resistance ratios ranging from 1.1- to 27.9-fold, indicating that the magnitude of resistance we detected (52.9-fold) is substantially higher than the typical levels reported in many Chinese rice-growing regions, likely reflecting more intensive and prolonged ALS herbicide selection pressure in Heilongjiang fields [25]. In Hubei Province, Gu et al. identified 15 resistant *E. crus-galli* populations with penoxsulam resistance, and the Trp-574-Leu mutation was detected in the majority (ten out of fifteen) of these resistant populations [26]. Notably, the in vitro ALS activity in three of their resistant populations was 51.28-, 5.51-, and 8.46-fold greater than that in the susceptible population, which is comparable to our finding of significantly enhanced ALS activity over 48 h following PSE treatment in our R population [26]. The W574L mutation (TGG-to-TTG substitution) we identified in our R population represents one of the most extensively documented ALS target-site substitutions conferring resistance across multiple chemical classes. This mutation has been reported to confer broad cross-resistance including SU, SU + SCT, and TP chemistries in grass weeds such as *Poa trivialis*, where resistance indices exceeding 214-fold have been reported for populations carrying W574L in combination with Pro-197 substitutions [27]. In *Poa annua* populations from Ireland, the W574L mutation, whether alone or in combination with Pro-197-Thr, was shown to confer high levels of resistance to SU, SU + SCT, and TP herbicides, with GR_50_ resistance indices exceeding 10-fold [28]. The consistent pattern across these studies underscores the critical role of the W574L mutation as a major driver of ALS inhibitor resistance across various grass weed species globally. However, the notable limitation of this study is the geographical separation between the S (Hebei Province) and R (Heilongjiang Province) populations. Although the S population was confirmed to be susceptible to PSE (with no prior exposure to ALS-inhibiting herbicides), genetic divergence resulting from geographical isolation may contribute to some phenotypic differences between the two populations that are not directly related to PSE resistance. For example, inherent differences in growth rates, metabolic capacity, or gene expression patterns between geographically distinct populations could potentially confound the interpretation of physiological and molecular differences attributed to resistance. Future studies should include a susceptible population collected from the same geographical region as the R population to eliminate this confounding factor and strengthen the causal link between observed differences and PSE resistance.

To investigate whether metabolic mechanisms might contribute to PSE resistance in the R population, we examined the effects of the CYP450 inhibitor malathion and the GST inhibitor NBD-Cl. Pretreatment with malathion reduced the GR_50_ of the R population by 98.7%, while NBD-Cl pretreatment resulted in a 98.4% reduction. These results provide indirect evidence suggesting possible involvement of both CYP450s and GSTs in resistance to PSE. Our findings with metabolic inhibitors are consistent with recent studies documenting elevated expression of detoxification genes in multi-resistant *E. crus-galli* populations [25]. However, it must be emphasized that inhibitor-based experiments provide only indirect evidence for metabolic resistance, as malathion and NBD-Cl may produce non-specific effects. Furthermore, because the S and R populations originated from geographically distinct locations (see detailed discussion below), it is possible that part of the differential inhibitor response reflects inherent population variation in basal metabolic enzyme activities rather than herbicide-induced resistance alone. Definitive confirmation of metabolic resistance will therefore require direct PSE metabolite profiling and functional characterization of candidate genes in isogenic or sympatric populations [29]. Our conclusion regarding the possible involvement of CYP450s and GSTs in PSE resistance is based solely on metabolic inhibitor experiments with malathion and NBD-Cl. Such experiments can provide important indications of metabolic resistance, but they do not constitute direct evidence of NTSR. Inhibitors may produce non-specific effects, and synergy does not unequivocally demonstrate enhanced herbicide metabolism [29]. Therefore, we present this work as suggestive of a possible role for metabolic resistance.

To further explore the molecular basis of target-site resistance, we employed molecular docking to investigate conformational changes in the ALS enzyme. Results suggest that resistance in the R population is associated with a significant reduction in binding affinity between ALS and PSE. This in silico analysis provides a structural hypothesis that may help explain the observed target-site resistance. The predicted alteration of the binding pocket is consistent with the reduced herbicide sensitivity observed in the R population; however, this in silico prediction provides a structural hypothesis rather than direct evidence for a causal mechanism and awaits experimental structural determination. Our docking results align with recent molecular modeling studies documenting the structural basis of ALS resistance. Zhao et al. reported that a Pro-197-Ser substitution in the ALS gene of resistant Vicia sativa populations reduced the binding energies between ALS active sites and tribenuron-methyl or florasulam by over 80%, rendering ALS enzymes about 17-fold less sensitive [30]. Kumar et al. provided structural insights into sulfonylurea herbicide resistance in wheat ALS, showing that single point substitutions at TaALS-P174S, double mutants (TaALS-G632S/P174S and TaALS-G631D/G632S), and a triple mutant conformation (TaALS-G631D/G632S/P174S) exhibited progressively lower binding affinity with the SU herbicide nicosulfuron compared to the wild-type TaALS [31]. Combined ALS activity assays and molecular docking in *Fimbristylis littoralis* further indicated that the Trp-574-Leu mutation substantially increases binding energy, reducing herbicide-ALS interaction [32].

In summary, this study provides four key demonstrated findings: (1) the *E. crus-galli* population from Heilongjiang exhibits high-level (52.9-fold) resistance to PSE; (2) the W574L ALS substitution is present in all sequenced individuals from the R population; (3) the mutant ALS enzyme shows significantly reduced sensitivity to PSE inhibition in vitro; and (4) pretreatment with CYP450 and GST inhibitors substantially restores PSE sensitivity in the R population. The most plausible interpretation of these results is that W574L-mediated TSR is the primary resistance mechanism, with a possible additional contribution from enhanced metabolism. The relative contribution of TSR and NTSR mechanisms remains unresolved and requires further investigation in genetically controlled backgrounds.

## 4. Materials and Methods

### 4.1. Chemicals and Plant Materials

PSE was obtained from Zhejiang Tianfeng Biotechnology Co., Ltd. (Jinhua, China) Malathion (70% emulsifiable concentrate) was obtained from Texas Green Dominance Fine Chemical Co., Ltd. (Dezhou, China). NBD-Cl (98%) was obtained from Shanghai Yuanye Biotechnology Co., Ltd. (Shanghai, China). Seeds of the PSE susceptible population (S) were collected from Tangshan City (39.29° N, 118.44° E), Hebei Province, China, where no ALS-inhibiting herbicides had been used before 2021. A total of 30 mature *E. crus-galli* plants were randomly sampled across a 0.5-ha field spaced at least 5 m apart to avoid sampling closely related individuals, and seeds were bulked to form the S population. The PSE resistant population (R) was collected from Baoqing City (46.33° N, 132.20° E), Heilongjiang Province, China, where PSE had failed to control weeds in 2021. Similarly, 30 plants were sampled from a 1.2-ha field with a 10-year history of continuous ALS-inhibitor use, and seeds were bulked. All seeds were air-dried at room temperature for 7 days, stored in paper bags at 4 °C in a dark refrigerator, and used within 6 months of collection. Species identity was confirmed by morphological characteristics (leaf sheath color, ligule shape, and inflorescence structure).

The total DNA of three-leaf *E. crus-galli* was extracted using a Total DNA Kit (DP305; Tiangen Biochemical Technology, Beijing, China). DNA concentrations were determined using a NanoDrop spectrophotometer (Gene Company, Hong Kong, China). Primers and the polymerase chain reaction (PCR) system and reaction conditions were similar to previous work [33]. Gene sequences were compared using SnapGene^®^ (Version 6.0.2).

### 4.2. Plant Material Cultivation and Herbicide Application

Uniform, disease-free, plump seeds were selected and subjected to germination assays. Fourteen healthy seeds were evenly placed in sterile Petri dishes (8.5 cm in diameter). Three independent biological replicates were prepared for each PSE concentration (S population for 0, 10, 20 and 30 ppm; R population for 0, 10, 100 and 500 ppm). The germination conditions were as follows: day/night temperatures of 28 °C and 22 °C, respectively; a photoperiod of 14 h light and 10 h dark; photosynthetic photon flux density maintained at 450 μmol m^−2^ s^−1^; and relative humidity kept constant at 75% throughout the culture period. Seed germination status was recorded daily at a fixed time, and the final germination rate for each treatment group was calculated after 14 consecutive days of culture.

Germinated seedlings with uniform growth vigor were selected and transplanted into pots for further whole-plant cultivation. The environmental parameters during pot culture were kept consistent with those of the germination-stage incubator to ensure uniform growth conditions for all experimental materials. Barnyard grass seedlings at the three-leaf stage (cultivated under uniform greenhouse conditions) were planted in pots containing sterilized moist loam soil (six vigorous and uniformly growing seedlings per pot). A 3WP-2000 walking-type spray tower (Nanjing Institute of Agricultural Mechanization, Ministry of Agriculture and Rural Affairs, Nanjing, China) equipped with a TP6501E flat-fan nozzle (TeeJet Technologies, Glendale Heights, IL, USA; 372 L hm^−2^, 390 mL min^−1^, 3.0 kgcm^−2^) was used for PSE treatment. The active ingredient application rates were set at 0, 7.5, 11.25, 22.5 and 67.5 g a.i. ha^−1^ for S population. As the R population exhibited high tolerance, 270 g a.i. ha^−1^ did not achieve 50% growth reduction; therefore, an extended range of 0, 22.5, 67.5, 450.0, 750.0, 1050.0, 1350.0 and 1650.0 g a.i. ha^−1^ was employed. Three independent biological replicates (individual pots) were established for each treatment.

### 4.3. Whole–Plant Dose–Response to PSE

Whole-plant dose–response bioassays were conducted using three-leaf-stage *E. crus-galli* seedlings grown in pots (six per pot) containing moist loam soil under glasshouse conditions. Plants were harvested at 21 days post-herbicide application. Only the aboveground parts (shoot tissues, excluding roots) of each seedling were collected to standardize sampling. From each biological replicate (pot), all six plants were harvested, and their aboveground fresh weight was averaged to give a single value per replicate. The relative fresh weight was then calculated based on the means of the three independent biological replicates. Thus, the pot served as the experimental unit in all statistical analyses, ensuring independence of observations. Aboveground fresh weight was recorded 21 days after application. Relative fresh weight data were calculated using the standard formula: Relative fresh weight (%) = (mean aboveground fresh weight of PSE-treated seedlings/mean aboveground fresh weight of blank solvent control seedlings) × 100%. All measurements were completed within 2 h of sampling to minimize weight loss from tissue dehydration.

### 4.4. Effect of Metabolic Enzyme Inhibitors on PSE Resistance

The effects of malathion (a CYP450 inhibitor) and NBD-Cl (a GST inhibitor) on PSE resistance were evaluated as described previously [6,34]. Malathion and NBD-Cl were prepared in 3% (*v*/*v*) aqueous acetone at concentrations equivalent to 1000 g a.i. ha^−1^ and 270 g a.i. ha^−1^, respectively. The following six treatment groups were established, each applied to the R population at the three-leaf stage: (1) untreated control (solvent only); (2) malathion alone; (3) NBD-Cl alone; (4) PSE alone (solvent pretreatment, followed by PSE at a series of rates); (5) malathion + PSE (malathion applied 2 h before PSE); and (6) NBD-Cl + PSE (NBD-Cl applied 48 h before PSE). For groups 4–6, PSE was applied at 0, 22.5, 67.5, 135.0, 202.5, and 270.0 g a.i. ha^−1^. Aboveground fresh weight was measured 21 days after PSE application and expressed as a percentage of the untreated control. Dose–response curves were fitted for groups 4–6 to derive GR_50_ values, and differences in GR_50_ between PSE alone and each inhibitor combination were assessed by the extra sum-of-squares F-test (*p* < 0.05). Relative fresh weights at selected PSE doses were compared by one-way ANOVA followed by Dunnett’s post-hoc test, with the PSE alone treatment group serving as the reference for inhibitor synergy assessment. GR_50_ values between PSE alone and each inhibitor combination were compared using the extra sum-of-squares F-test.

### 4.5. Target-Site Gene Sequencing

Genomic DNA was extracted from *E. crus-galli* seedlings using a Plant Genomic DNA Kit (Tiangen Biotech Co., Ltd., Beijing, China) following the manufacturer’s protocol. The ALS gene fragment encompassing all known resistance-conferring mutation sites was amplified from the S and R populations using gene-specific primers (listed in Table S8). PCR amplification was performed in a 25 μL reaction volume containing 1 μL of genomic DNA, 0.4 μM of each primer, and 12.5 μL of 2 × Taq Plus Master MIX II (TransGen Biotech, Beijing, China). The PCR cycling conditions are detailed in Table S9. Amplified products were sequenced and analyzed for mutations. For both S and R populations, 10 independent plants were selected for ALS gene sequencing, with three PCR amplifications performed per plant. Three clones from each amplification product were selected for sequencing to verify mutation consistency.

### 4.6. ALS Activity Assay

In vitro ALS activity assays were conducted using three-leaf-stage seedlings of the S and R *E. crus-galli* populations. At time zero, plants were treated with PSE at 22.5 g a.i. ha^−1^ using the spray equipment described in Section 2.2. Leaf tissue (approximately 0.5 g fresh weight per sample) was harvested at 0 (immediately before spraying), 12, 24, 36, and 48 h after PSE application. Each sample was immediately frozen in liquid nitrogen and stored at −80 °C until enzyme extraction. For each population and each time point, three independent biological replicates (separate pots) were collected.

Collected fresh leaf tissue was homogenized in extraction buffer (100 mmol L^−1^ potassium phosphate, pH 7.5, 10 mmol L^−1^ sodium pyruvate, 5 mmol L^−1^ MgCl_2_, 1 mmol L^−1^ thiamine pyrophosphate, 10 μmol L^−1^ flavin adenine dinucleotide (FAD), and 10% glycerol). After centrifugation at 12,000 rpm for 10 min at 4 °C, the supernatant was used for the ALS reaction. All reagents were pre-warmed in a 25 °C water bath for 10 min before use. The assay mixture contained 50 mmol L^−1^ potassium phosphate (pH 7.0), 40 mmol L^−1^ sodium pyruvate, 10 mmol L^−1^ MgCl_2_, 1 mmol L^−1^ thiamine pyrophosphate, 10 μmol L^−1^ FAD, and enzyme extract in a total volume of 500 μL. The reaction was terminated by adding 50 μL of 6 mol L^−1^ H_2_SO_4_, followed by incubation at 60 °C for 15 min to decarboxylate acetolactate to acetoin. ALS activity was expressed as mg acetoin produced per gram fresh weight per hour (mg g^−1^ h^−1^).

Acetoin formation was quantified colorimetrically using a modified Westerfeld method. To each terminated reaction, 500 µL of 0.5% (*w*/*v*) creatine and 500 µL of 5% (*w*/*v*) α-naphthol (prepared in 2.5 mol L^−1^ NaOH) were added. The mixture was incubated at 60 °C for 15 min to develop the red color, then centrifuged at 5000 rpm for 5 min to remove precipitates. Absorbance was measured at 525 nm using a spectrophotometer. Negative controls (reaction mixture without enzyme extract) and background controls (enzyme extract added after acid termination) were included in every assay batch to account for non-enzymatic acetoin formation. A standard curve was prepared using authentic acetoin (0–200 µg mL^−1^) processed identically. ALS activity was calculated as:

$$\mathrm{ALS}\;\mathrm{activity}\;(\mathrm{mg}\;g^{-1}\;h^{-1}\;\mathrm{FW})\;=\;\frac{\mathrm{Acetoin}\;\mathrm{produced}\;(\mathrm{mg})}{\mathrm{Fresh}\;\mathrm{weight}\;\mathrm{of}\;\mathrm{extracted}\;\mathrm{tissue}\;(g)\;\times \;\mathrm{Reaction}\;\mathrm{time}\;(h)}$$

where the amount of acetoin was determined from the standard curve.

Data from the ALS activity time-course experiment were analyzed by two-way analysis of variance (ANOVA), with population (S vs. R) and time (0, 12, 24, 36, and 48 h) as fixed factors. When the population × time interaction was significant, pairwise comparisons between S and R populations at each time point were conducted using Tukey’s Honestly Significant Difference (HSD) post-hoc test, controlling the familywise error rate across all five time points (0, 12, 24, 36, and 48 h). Statistical significance was accepted at *p* < 0.05.

### 4.7. Molecular Docking of ALS with PSE and Structure-Activity Relationships

Two ALS crystal structures were used as templates for homology modeling: 3E9Y (complexed with the sulfonylurea herbicide monosulfuron) and 1Z8N (complexed with the imidazolinone herbicide imazaquin). Both structures were retrieved from the RCSB Protein Data Bank using Swiss-Model (PDB DOI: https://doi.org/10.2210/pdb3E9Y/pdb for 3E9Y; https://doi.org/10.2210/pdb1Z8N/pdb for 1Z8N) [35]. The *E. crus-galli* ALS amino acid sequence shared 75.13% identity with the Arabidopsis template over 88% coverage, indicating a high-quality homology model suitable for molecular docking studies. The quality of the homology models was assessed by multiple tools. ERRAT scores were 95.2339 and 91.8728 in the S and R population ALS models, respectively. And Ramachandran plot analysis showed that 92.4% and 91.0% of residues in the S and R population ALS models, respectively, were in the most favored regions, with no residues in disallowed regions (Table S10) [7]. For protein preparation, water molecules and heteroatoms were removed from the modeled structures, polar hydrogen atoms were added, and Gasteiger partial charges were assigned using AutoDock Tools. The final prepared protein structures were then saved in PDBQT format. The three-dimensional structure of PSE was obtained from PubChem (SMILES: CCOC(=O)C1=C(N(N=C1)C)S(=O)(=O)NC(=O)NC2=NC(=CC(=N2)OC)OC) and served as the ligand. Ligand structure was then prepared for docking by adding Gasteiger charges and saving in PDBQT format.

Molecular docking and optimizations were performed using the AutoDock Vina 1.2.7 version [36,37]. The dimensions of the docking box were set at 22.1 Å × 21.6 Å × 23.0 Å with a grid spacing of 1.0 Å, centered at coordinates x = 61.9 Å, y = 45.1 Å, z = 138.8 Å to cover the herbicide-binding pocket. The exhaustiveness was set to 10, and 16 binding poses were generated for each docking run. The pose with the lowest binding energy was selected as the representative binding mode. The docking protocol was validated by re-docking the co-crystallized ligand (monosulfuron) into the active site of the template structure (PDB: 3E9Y), achieving a root-mean-square deviation (RMSD) of 2.14 Å, indicating that the docking parameters reliably reproduce the experimental binding mode.

### 4.8. Data Analysis

All statistical analyses were performed using R version 4.6.0 [38]. Dose–response curves were fitted with the four-parameter log-logistic model using the drc package [35]. The extra sum-of-squares F-test was used to compare GR_50_ values between populations (S vs. R) and between treatments (PSE alone vs. PSE with malathion or PSE with NBD-Cl), with *p* < 0.05 considered statistically significant [39]. Two-way ANOVA followed by Tukey’s HSD test (for ALS activity time courses), one-way ANOVA followed by Dunnett’s test (for metabolic inhibitor experiments) were conducted using base R and the multcomp package [40]. The complete ANOVA tables for all F-tests and two-way ANOVA are provided in Supplementary Tables S1, S4, S5 and S11.

GR_50_ values were derived by fitting a four-parameter log-logistic regression model [41]:

*Y* = *c* + (*d* − *c*)/[1 + (*x*/GR_50_)*^b^*]

where, *Y* indicates the percentage of fresh weight; *c* and *d* are the lower and upper limits of the dosages, respectively; *x* is the PSE dose; and *b* is the slope of the *GR*_50_ curve. In turn, the level of herbicide resistance was calculated as a resistance index (RI) according to the following equation:

$$\mathrm{RI}\;=\;{\mathrm{GR}}_{50}(R)/{\mathrm{GR}}_{50}\left(S\right)$$

Resistance levels were classified based on RI values according to national guidelines (NY/T 2728–2015) susceptible (S, RI ≤ 1), low resistance (LR, 1 < RI ≤ 3), intermediate resistance (MR, 3 < RI ≤ 10), and high resistance (HR, RI > 10).

## 5. Conclusions

This study identified a highly PSE-resistant *E. crus-galli* population from Heilongjiang Province and associated this phenotype with the ALS W574L substitution, supported by ALS gene sequencing, ALS activity assays and molecular docking predictions. Inhibitor bioassays with malathion and NBD-Cl further suggest that CYP450- and GST-related metabolic processes may contribute to resistance, although direct metabolic evidence is still required. Because the susceptible and resistant populations originated from different geographic regions, future studies should include geographically matched susceptible populations, direct herbicide metabolism assays, expression analyses of candidate detoxification genes and functional validation of the W574L substitution. These additional analyses would clarify the relative contribution of TSR and NTSR mechanisms and support more robust integrated management strategies for ALS-inhibitor-resistant *E. crus-galli* in rice production systems.

## Figures and Tables

**Table 1:** GR_50_ Values and RIs of *E. crus-galli* populations based on the four-parameter Log-Logistic Equation.

Population	Regression Parameters				GR_50_ ± SE (g a.i. ha^−1^)	95% CI	RI	*p* Value ^†^
	c ± SE	d ± SE	b ± SE	r^2^				
S	−2.59 ± 2.70	97.73 ± 2.18	3.89 ± 0.49	0.992	12.32 ± 0.45	11.40–13.24	—	—
R	22.00 ± 4.36	91.50 ± 1.52	3.22 ± 0.63	0.965	651.76 ± 43.95	562.70–740.82	52.91	<0.0001

^†^ Extra sum-of-squares F-test comparing GR_50_ values between S and R populations (Table S1). 95% CI, 95% confidence interval of GR_50_.

**Table 2:** GR_50_ Values and RIs of R populations under PSE with CYP450s and GSTs inhibitors based on the four-parameter Log-Logistic Equation.

Treatment	Regression Parameters				GR_50_ ± SE (g a.i. ha^−1^)	95% CI	*p* Value ^†^
	c ± SE	d ± SE	b ± SE	r^2^			
PSE alone	22.00 ± 4.36	91.50 ± 1.52	3.22 ± 0.63	0.965	651.76 ± 43.95	562.70–740.82	—
PSE + Malathion	30.38 ± 0.90	98.35 ± 1.20	3.65 ± 0.42	0.992	8.88 ± 0.23	8.41–9.36	<0.0001
PSE + NBD-Cl	30.73 ± 0.74	99.28 ± 0.94	2.76 ± 0.21	0.995	10.51 ± 0.26	9.97–11.06	<0.0001

^†^ Extra sum-of-squares F-test comparing GR_50_ values between PSE alone and each inhibitor treatment (Tables S4 and S5).

**Table 3:** ALS activity (mg g^−1^ h^−1^ FW) in S and R *E. crus-galli* populations after PSE treatment (22.5 g a.i. ha^−1^).

Time (h)	S Population (Mean ± SE) (mg g^−1^ h^−1^ FW)	R Population (Mean ± SE) (mg g^−1^ h^−1^ FW)	*p*-Value ^†^	Significance
0	20.67 ± 0.33	25.67 ± 0.33	0.0295	*
12	17.67 ± 0.88	24.00 ± 0.58	0.0033	**
24	12.33 ± 0.88	25.00 ± 1.15	<0.0001	***
36	12.67 ± 0.33	22.00 ± 1.15	<0.0001	***
48	11.33 ± 0.88	23.00 ± 1.73	<0.0001	***

^†^ *p*-values from Tukey’s HSD post-hoc test following two-way ANOVA (Table S6). Asterisks indicate significant differences between S and R populations at each time point by Tukey’s HSD test: * *p* < 0.05, ** *p* < 0.01, *** *p* < 0.001.

## Data Availability

The ALS gene sequences from the S and R populations are provided in Supplementary Tables S9 and S10. All other primary data supporting the conclusions of this study, including complete dose–response data tables, numerical values underlying all figures, and the molecular docking PDB files, are provided as Supplementary Datasets. Any further inquiries can be directed to the corresponding authors (sdlxj818@163.com (X.L.) and dshengli@aliyun.com (S.D.)).

## Supplementary Materials

The following supporting information can be downloaded at: https://www.mdpi.com/article/10.3390/plants15111611/s1, Figure S1: Survival rate of S and R populations under different PSE dosages; Figure S2: Plant height of S and R populations under different PSE dosages; Figure S3: Relative dry weight of S and R populations under different PSE dosages; Table S1: Extra sum-of-squares F-test for GR_50_ comparison between S and R populations; Table S2: Near full-length ALS sequences from S population individuals; Table S3: Near full-length ALS sequences from R population individuals; Table S4: Extra sum-of-squares F-test for GR_50_ comparison between PSE alone and PSE + Malathion; Table S5: Extra sum-of-squares F-test for GR_50_ comparison between PSE alone and PSE + NBD-Cl; Table S6: Complete two-way ANOVA table for ALS enzyme activity data; Table S7: Predicted intermolecular interactions between PSE and the ALS active sites in the S and R populations of *E. crus-galli*; Table S8: PCR primers used in the ALS gene cloning and sequencing; Table S9: PCR conditions used in this study; Table S10: Homology model quality metrics of ALS in *E. crus-galli*; Table S11: Two-way ANOVA table for relative dry weight data.
