# Supplementary material for: Mechanism of Echinochloa crus-galli Resistance to the ALS-Inhibiting Herbicide Pyrazosulfuron-ethyl in China
Source: Plants (Basel). 2026 May 24;15(11):1611. doi: 10.3390/plants15111611 (PMC13258948; doi:10.3390/plants15111611)
Supplement: Supplementary file 1 [file plants-15-01611-s001.zip › plants-4266359-supplementary.pdf]

Supplementary Data for

Mechanism of *Echinochloa crus-galli* Resistance to the ALS-Inhibiting Herbicide Pyrazosulfuron-ethyl in China

Qing Liu [1], Rongxue Zhang [2,3], Linjing Sun [2,3], Xin Lu [2,3], Gaoping Xu [2,3], Hui Tong [2,3], Binglei Zhang [2,3], Xuejun Liu [2,3,*] and Shengli Du [1,4,5,*]

1 College of Life Sciences, Nankai University, Tianjin 300071, China
2 Modern Agricultural Science and Technology Research Institute, Tianjin Agricultural University, Tianjin 300392, China
3 Tianjin Key Laboratory of Crop Genetics and Breeding, Tianjin Crop Research Institute, Tianjin 300392, China
4 Cucumber Research Institute, Tianjin Academy of Agricultural Sciences, Tianjin 300192, China
5 State Key Laboratory of Vegetable Biobreeding, Tianjin 300192, China
* Correspondence: sdlxj818@163.com (X.L.); dshengli@aliyun.com (S.D.)

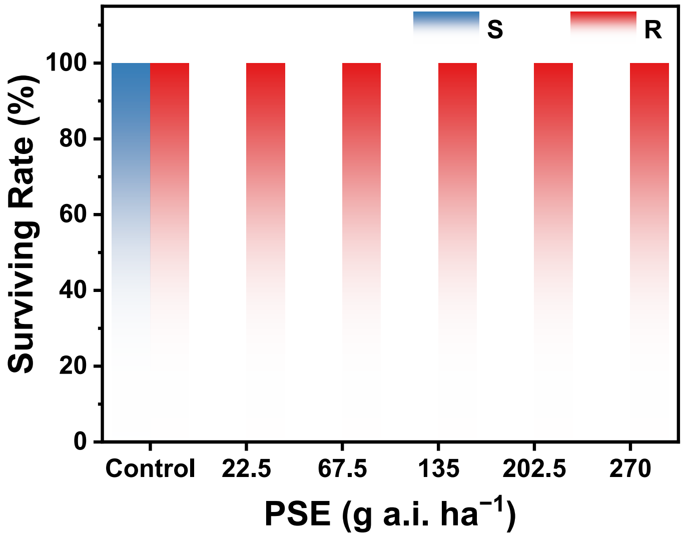

Figure S1. The surviving rate of S and R populations under different dosages.

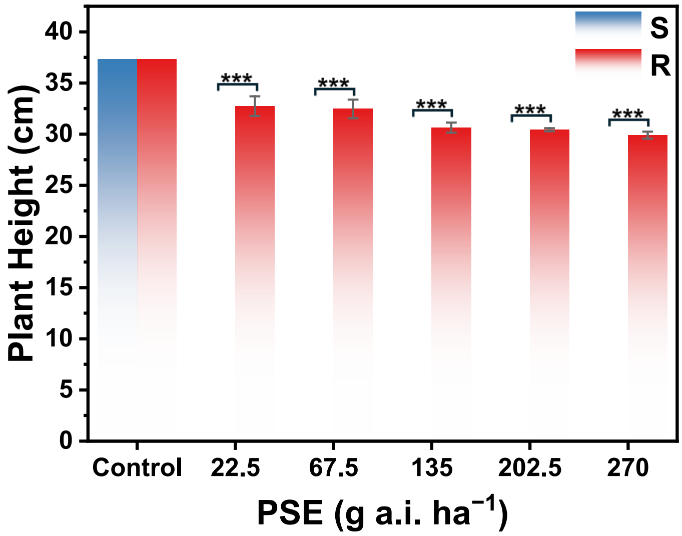

Figure S2. The plant height of S and R populations under different dosages. Asterisks represent significant differences (***P < 0.001).

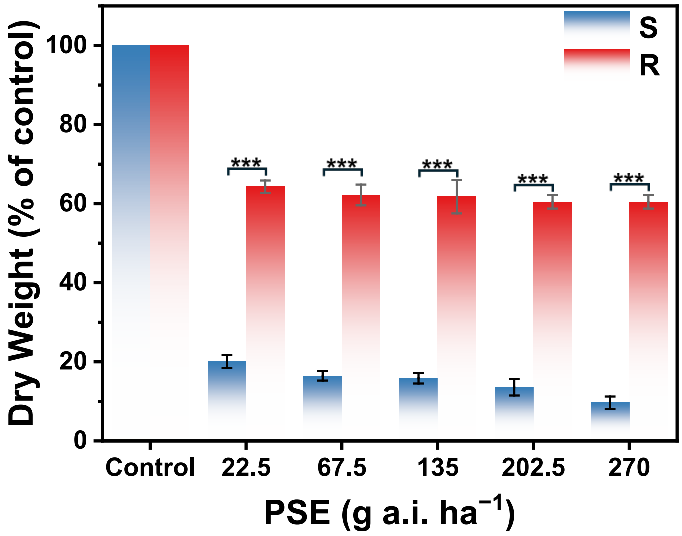

Figure S3. The relatively dry weight of S and R populations under different dosages. Asterisks represent significant differences (***P < 0.001).

Table S1. Extra sum-of-squares F-test for GR_{50} comparison between S and R populations.

Model	Res. Df	RSS	Df	F value	P value
Reduced (shared GR_{50})	48	1011.2	—	—	—
Full (separate GR_{50})	47	1011.2	1	227.95	<0.0001

Note: Res. Df represents residual degrees of freedom; RSS represents residual sum of squares.

Table S2. Near full-length ALS sequences from S population individuals.

MAAATTTTTTSSSISFSTKPSPSSSKSPLPISRFSLPFSLNPNKSSSSSRRRGIKSSSPSSISAVLNTTTNVTTTP
SPTKPTKPETFISRFAPDQPRKGADILVEALERQGVETVFAYPGGASMEIHQALTRSSSIRNVLPRHEQG
GVFAAEGYARSSGKPGICIATSGPGATNLVSGLADALLDSVPLVAITGQVPRRMIGTDAFQETPIVEVT
RSITKHNYLVMDVEDIPRIIEEAFFLATSGRPGPVLVDVPKDIQQQLAIPNWEQAMRLPGYMSRMPKP
PEDSHLEQIVRLISESKKPVLYVGGGCLNSSDELGRFVELTGIPVASTLMGLGSYPCDDELSLHMLGMH
GTVYANYAVEHSDLLLAFGVRFDDRVTGKLEAFASRAKIVHIDIDSAEIGKNKTPHVSVCGDVKLALQ
GMNKVLENRAEELKLDFGVWRNELNVQKQKFPLSFKTFGEAIPPQYAIKVLDELTDGKAIISTGVGQ
HQMWAAQFYNYKKPRQWLSSGGLGAMGFGLPAAIGASVANPDAIVVDIDGDGSFIMNVQELATIR
VENLPVKVLLLNNQHLGMVVQWEDRFYKANRAHTFLGDPAQEDEIFPNMLLFAAACGIPAARVTK
KADLREAIQTMLDTPGPYLLDVICPHQEHVLPMIPSGGTFNDVITEGDGRIKY

Table S3. Near full-length ALS sequences from R population individuals.

MAAATTTTTTSSSISFSTKPSPSSSKSPLPISRFSLPFSLNPNKSSSSSRRRGIKSSSPSSISAVLNTTTNVTTTP
SPTKPTKPETFISRFAPDQPRKGADILVEALERQGVETVFAYPGGASMEIHQALTRSSSIRNVLPRHEQG
GVFAAEGYARSSGKPGICIATSGPGATNLVSGLADALLDSVPLVAITGQVPRRMIGTDAFQETPIVEVT
RSITKHNYLVMDVEDIPRIIEEAFFLATSGRPGPVLVDVPKDIQQQLAIPNWEQAMRLPGYMSRMPKP
PEDSHLEQIVRLISESKKPVLYVGGGCLNSSDELGRFVELTGIPVASTLMGLGSYPCDDELSLHMLGMH
GTVYANYAVEHSDLLLAFGVRFDDRVTGKLEAFASRAKIVHIDIDSAEIGKNKTPHVSVCGDVKLALQ
GMNKVLENRAEELKLDFGVWRNELNVQKQKFPLSFKTFGEAIPPQYAIKVLDELTDGKAIISTGVGQ
HQMWAAQFYNYKKPRQWLSSGGLGAMGFGLPAAIGASVANPDAIVVDIDGDGSFIMNVQELATIR
VENLPVKVLLLNNQHLGMVVQLEDRFYKANRAHTFLGDPAQEDEIFPNMLLFAAACGIPAARVTKK
ADLREAIQTMLDTPGPYLLDVICPHQEHVLPMIPSGGTFNDVITEGDGRIKY

Table S4. Extra sum-of-squares F-test for GR_{50} comparison between PSE alone and PSE + Malathion.

Model	Res. Df	RSS	Df	F value	P value
Reduced (shared GR_{50})	47	16132	—	—	—
Full (separate GR_{50})	43	922	4	177.33	<0.0001

Table S5. Extra sum-of-squares F-test for GR_{50} comparison between PSE alone and PSE + NBD-Cl.

Model	Res. Df	RSS	Df	F value	P value
Reduced (shared GR_{50})	47	14290.7	—	—	—
Full (separate GR_{50})	43	852.9	4	169.38	<0.0001

Table S6. Complete two-way ANOVA table for ALS enzyme activity data.

Source	Df	Sum Sq	Mean Sq	F value	P value
Population	1	607.5	607.5	233.65	<0.001
Time	4	156.2	39.1	15.02	<0.001
Population × Time	4	65.7	16.4	6.31	0.0019
Residuals	20	52.0	2.6		

Table S7. Predicted intermolecular interactions between PSE and the ALS active sites in the S and R populations of *E. crus-galli*.

Population	Predicted binding energy (kcal·mol^{-1})	ALS residue	Interaction type	Predicted distance (Å)	Ligand atom involved
S	−6.61	Ser-168	Hydrogen bond	3.21	O
		Phe-206	Hydrophobic (π-alkyl)	3.74	Pyrazole ring
		Gln-207	Hydrogen bond	2.54	N
		Lys-256	Hydrogen bond	1.67	O
		Lys-256	Salt bridge	5.35	Carboxylate
		Gln-260	Hydrogen bond	2.97	N
		Arg-377	Hydrogen bond	2.58	O
		Arg-377	Salt bridge	5.42	Carboxylate
		Trp-574	Hydrophobic (π-π stacking)	3.69	Pyrimidine ring
		Ser-653	Hydrogen bond	2.00	N
R (W574L mutant)	−4.61	Asp-576	Hydrogen bond	1.89	O
		Arg-577	Hydrogen bond	2.13	O
		His-646	Hydrogen bond	3.41	O
		Asp-665	Hydrogen bond	2.24	N

Table S8. PCR primers used in the ALS gene cloning and sequencing.

Primer	Sequence (5′–3′)	References
BYG12-F	GCAAGGGCGCCGACATCCT	[17]
BYG12-R	CCTGCTTGCAAAAGCCTCAAT	
BYG22-F	ATTGAGGCTTTTGCAAGCAGG	
BYG22-R	ATACACAGTCCTGCCATCACC	
BYG31-F	AAGGACATCCAGCAGCAGAT	
BYG31-R	TGAAGACAACCACTGCCTTG	

Table S9. PCR conditions used in this study.

Gene	Primer	Codon	Fragment length(bp)	Annealing	Denaturing	Elongation	No Cycles
ALS	BYG12-F<br>BYG12-R	122,197,205,206,256,376,377	875	95℃(15s)	59℃(15s)	72℃(40s)	35
	BYG22-F<br>BYG22-R	574,653,654	867	95℃(15s)	59℃(15s)	72℃(40s)	35
	BYG31-F<br>BYG31-R	256,376,377	756	95℃(15s)	58℃(15s)	72℃(40s)	35

Table S10. Homology model quality metrics of ALS in *E. crus-galli*.

**Model**	**ERRAT**	**Ramachandran favored (%)**	**Ramachandran outliers (%)**
S population ALS (WT)	95.2339	92.4	0.0
R population ALS (W574L)	91.8728	91.0	0.0

Table S11. Two-way ANOVA table for relative dry weight data.

Source	Df	Sum Sq	Mean Sq	F value	P value
Source	Df	Sum Sq	Mean Sq	F value	P value
Population	1	18520.3	18520.3	12570.0	<0.001
Dose	4	32.1	8.0	5.44	0.0022
Population × Dose	4	138.5	34.6	23.50	<0.001

References

17. Feng, T.; Peng, Q.; Wang, L.; Xie, Y.; Ouyang, K.; Li, F.; Zhou, H.; Ma, H., Multiple resistance mechanisms to penoxsulam in *Echinochloa crus-galli* from China. Pestic. Biochem. Physiol. 2022, 187, 105211.
